# Epitranscriptomic alterations induced by environmental toxins: implications for RNA modifications and disease

**DOI:** 10.1186/s41021-025-00337-9

**Published:** 2025-08-04

**Authors:** Esther Ugo Alum, Regina Idu Ejemot-Nwadiaro, Mariam Basajja, Daniel Ejim Uti, Okechukwu Paul-Chima Ugwu, Patrick Maduabuchi Aja

**Affiliations:** 1 Department of Research and Publications, Kampala International University, P. O. Box 20000, Kampala, Uganda; 2 Directorate of Research, Innovation, Consultancy and Extension (RICE), Kampala International University, Kampala, Uganda; 3 Leiden Institute of Advanced Computer Science (LIACS), Leiden University, Leiden, Netherlands; 4 Department of Biochemistry, Kampala International University, P. O. Box 20000, Kampala, Uganda; 5 Department of Biochemistry, Faculty of Basic Medical Sciences, Federal University of Health Sciences, Otukpo, Benue State Nigeria

**Keywords:** Epitranscriptomics, RNA modifications, Environmental toxins, M6A, M5C, Pseudouridine, Biomarker discovery, Cancer diagnosis, High-throughput sequencing, Therapeutic strategies

## Abstract

Epitranscriptomics, the study of chemical modifications on RNA molecules, has emerged as a vital regulatory layer in gene expression. Among the numerous RNA modifications, N6-methyladenosine (m6A), 5-methylcytosine (m5C), and pseudouridine (Ψ) are particularly significant in maintaining RNA stability, splicing, translation, and cellular function. This review specifically explores how environmental toxins such as heavy metals, air pollutants, pesticides, and endocrine-disrupting chemicals can disrupt RNA modifications, leading to pathophysiological consequences. By focusing on major RNA marks and their associated regulatory enzymes (writers, erasers, and readers), we elucidate the molecular mechanisms through which toxins impair RNA processing and translation. These disruptions have been increasingly linked to disease development, including cancer, neurodegenerative disorders, metabolic syndromes, and immune dysfunction. Moreover, the review highlights recent technological advances such as high-throughput sequencing, mass spectrometry, and CRISPR-based RNA editing that enable detection and characterization of epitranscriptomic changes. Importantly, RNA modifications are dynamic and reversible, making them promising candidates for use as early biomarkers of environmental exposure and disease risk. We also discuss emerging non-canonical RNA modifications and their roles in toxicological responses. The potential of RNA-based biomarkers to enhance environmental health monitoring and precision medicine is significant, although challenges such as methodological standardization, context-specific variability, and clinical translation remain. This synthesis offers a focused, mechanistic perspective on the intersection of RNA biology and environmental toxicology, emphasizing the relevance of RNA modifications as both molecular targets and diagnostic tools in assessing the biological impact of environmental insults.

**Graphical Abstract:**

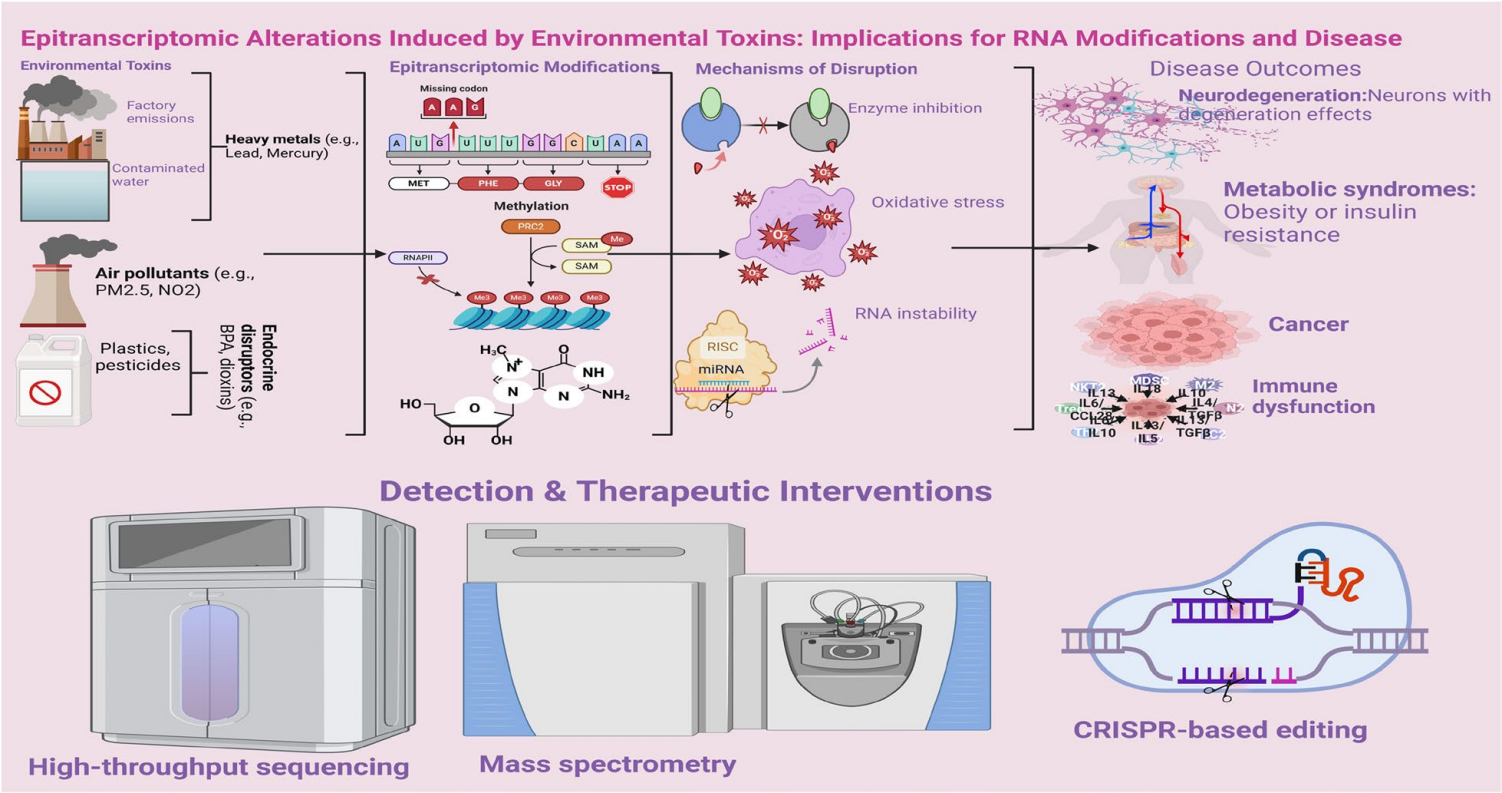

## Introduction

Epitranscriptomics, the study of chemical modifications on RNA, has emerged as a critical layer of gene regulation in modern molecular biology [1]. This studies the post-transcriptional RNA changes affecting gene expression without changing the fundamental nucleotide sequence [1]. These modifications, more than 170 identified to date play a pivotal role in post-transcriptional control of gene expression by influencing RNA stability, splicing, transport, localization, and translation [1]. Among the most studied modifications are N6-methyladenosine (m6A), 5-methylcytosine (m5C), pseudouridine (Ψ), N1-methyladenosine (m1A), and 2′-O-methylation (Nm) [2, 3]. These modifications regulate cellular homeostasis and are dynamically written, read, and erased by specific RNA-modifying enzymes [4]. They are now recognized as central regulators in diverse physiological processes and pathological conditions, including cancer, neurodegenerative disorders, metabolic dysfunctions, and immune regulation [5].

While the field has advanced considerably in defining the biological functions of RNA modifications, a growing but underexplored area is their vulnerability to environmental toxicants. Chemical pollutants including heavy metals like arsenic, cadmium, and lead; air pollutants such as particulate matter (PM2.5 and PM10); and endocrine-disrupting compounds like bisphenol A and phthalates can interfere with the epitranscriptome [6]. These compounds can either directly inhibit the activity of RNA-modifying enzymes or induce oxidative stress, leading to abnormal RNA modification patterns. As a result, altered RNA methylation and structural changes can disrupt gene expression and cellular function, acting as an early molecular mechanism of environmental disease pathogenesis [7, 8].

This review specifically examines how environmental toxins modify key RNA alterations such as m6A, m5C, Ψ, m1A, and Nm. It investigates how these modifications are dysregulated by external toxicants and explores the downstream effects on disease development. The aim is to define the molecular pathways through which toxins influence RNA-modifying enzyme expression and activity, destabilize RNA-protein interactions, and ultimately contribute to diverse pathological outcomes. By establishing this mechanistic link, the review brings needed clarity to a relatively nascent but significant research niche at the intersection of environmental toxicology and RNA biology.

Focusing on RNA modifications offers distinct advantages over traditional molecular markers of exposure and disease. Unlike DNA mutations, which are permanent, RNA modifications are reversible and dynamically regulated [9]. This makes them highly responsive to environmental stimuli and excellent candidates for real-time biomarkers of cellular stress [9]. For example, exposure to arsenic suppresses METTL3, an m6A “writer,” reducing methylation of tumor suppressor transcripts and promoting carcinogenesis [10]. Cadmium disrupts tRNA m5C methylation, impairing protein synthesis and increasing organ-specific toxicity. Air pollutants reduce pseudouridine stability, leading to defects in RNA structure and translation [11]. These examples reflect not only the specificity of environmental effects on RNA chemistry but also the potential utility of RNA modifications as early indicators of toxin exposure and disease risk.

The biological consequences of such epitranscriptomic alterations are far-reaching. Aberrant RNA methylation or loss of RNA structural integrity is increasingly recognized as a driving factor in oncogenesis, neurodegeneration, metabolic dysregulation, and immune impairment [7]. For instance, altered m6A methylation can enhance the translation of oncogenic mRNAs or suppress the stability of tumor suppressors [12]. In neurological disorders, oxidative stress-induced pseudouridine loss disrupts synaptic function [13]. In metabolic diseases, endocrine disruptors alter m1A and m6A profiles in insulin-related genes, contributing to glucose imbalance and obesity [14]. These findings suggest that RNA modifications function not merely as passive readouts of environmental stress but as active mediators of disease phenotypes.

This emerging understanding is further empowered by rapid technological advances. High-throughput sequencing technologies such as MeRIP-seq and Pseudo-seq, as well as mass spectrometry and CRISPR-based RNA editing tools, have enabled precise mapping and quantification of RNA modifications [15, 16]. These innovations allow researchers to detect environmental toxin-induced changes in RNA in both coding and non-coding regions, identify disease-linked modification patterns, and design molecular interventions. The field is now positioned to transition from discovery-based research to translational applications, including diagnostic biomarker development and targeted therapeutics. Despite its promise, several challenges limit the full integration of epitranscriptomic analysis into environmental and clinical sciences [17]. These include the lack of standardized protocols for RNA modification detection, limited validation of biomarkers in human populations, and interindividual variability in response to toxin exposure [18, 19]. Furthermore, RNA modification patterns are often cell-type-specific and temporally dynamic, requiring sensitive and context-aware analytical platforms [20]. Addressing these barriers will be essential for establishing RNA modifications as reliable biomarkers for environmental health surveillance and disease risk stratification.

Taken together, this review critically explores the role of RNA modifications as molecular targets and biomarkers of environmental toxin exposure. By highlighting mechanistic links between chemical exposures and specific RNA modifications, the review provides a focused and integrative synthesis that contributes new insights into toxicogenomics, molecular pathology, and precision medicine. The knowledge of how environmental insults reshape the RNA modification landscape is not only essential for deciphering disease etiology but also opens novel avenues for diagnostics, risk prediction, and therapeutic intervention in an increasingly polluted world.

## Research methodology

Multiple scientific databases including PubMed, Scopus, Web of Science, and Google Scholar were searched comprehensively in this narrative review study. Studies published in peer-reviewed journals, conference proceedings, and reliable publications on environmental toxins and epitranscriptomics governed the search. Some of the words that were searched for were “epitranscriptomic modifications,” “RNA methylation,” “m6A, m5C, pseudouridine alterations,” “heavy metals and RNA modifications,” “air pollutants and RNA methylation,” “toxin-induced RNA dysregulation,” and “non-coding RNA and toxicology.” Peer-reviewed publications applying experimental, computational, or clinical methodologies to explore toxin-induced epitranscriptomic changes published in English within the last 15 years (2010–2025) were selected to ensure the recency and relevance of the review. Including several sources helped reduce bias by avoiding overreliance on one study and also comparing the results of several research approaches. To improve flow and readability, the findings were concurrently presented and discussed in several short sections.

## Overview of selected epitranscriptomics (RNA) modifications

RNA modifications represent an essential layer of post-transcriptional regulation, modulating RNA stability, translation, splicing, and degradation [21]. These dynamic and reversible chemical marks act as cellular sensors to environmental changes, including chemical exposures. The five major RNA modifications most extensively studied in relation to environmental toxins include m6A, m5C, pseudouridine (Ψ), N1-methyladenosine (m1A), and 2′-O-methylation (Nm) [7, 22]. Each modification plays distinct but overlapping roles in RNA metabolism and is linked to disease phenotypes when dysregulated, particularly under the influence of toxicants [7]. The understanding of these modifications is central to unraveling the epitranscriptomic mechanisms of environmental pathophysiology. Three functional groups define RNA modifications: methylation-based modifications, which add methyl groups to RNA nucleotides; isomerisation modifications, which change nucleotide structure without changing base pairing properties; and sugar and backbone modifications, which impact ribose sugar or phosphate backbone [23].

### Effects of environmental chemicals on RNA modifications

#### N6-Methyladenosine (m6A) modification

Recent studies on m6A modification have demonstrated its essential role in the control of post-transcriptional gene expression and many biological processes [24]. m6A is the most abundant internal modification in eukaryotic mRNA, influencing splicing, stability, and translation efficiency. As the predominant RNA epigenetic modification, m6A modulates mRNA metabolism via writers, erasers, and readers, impacting processes such as cancer and lipid metabolism [24]. M6A is deposited by methyltransferase complexes comprising METTL3, METTL14, and WTAP (writers), demethylated by FTO and ALKBH5 (erasers), and identified by proteins including YTHDF and IGF2BP families (readers) for dynamic control [25]. Research has revealed m6A’s dual function in influencing chromatin dynamics and transcriptional output, with m6A-modified chromatin-associated RNAs serving as molecular docking sites for histone modification proteins [26]. Exposure to heavy metals (e.g., arsenic and cadmium) and air pollutants (e.g., PM2.5), Aflatoxins, and cigarette smoking has been shown to alter m6A levels, disrupting normal RNA metabolism and contributing to diseases like cancer and neurodegeneration [27]. This alteration occurs in both coding and non-coding RNAs, significantly influencing cancer progression and therapeutic response [28]. The growing investigation into m6A alteration has revealed new possibilities in neuroscience, developmental biology, and oncology, underscoring its promise as a therapeutic target for cancer treatment [26, 28].

The mechanistic understanding of how environmental toxicants alter RNA modifications is of great importance, and it is important to move beyond general descriptors and discuss specific chemical constituents and their pathways of action. For instance, in tobacco smoke, benzo[a]pyrene (BaP) is a polycyclic aromatic hydrocarbon that forms DNA and RNA adducts. Studies show that BaP induces oxidative stress, which in turn impairs m6A methylation by suppressing METTL3 expression and altering reader protein (e.g., YTHDF2) localization, ultimately destabilizing oncogene transcripts. This contributes to the increased translational efficiency of oncogenic mRNAs in lung epithelial cells [29–31]. In the case of aflatoxins, particularly aflatoxin B1 (AFB1), the toxin interferes with the catalytic activity of RNA methyltransferases, including NSUN2 and METTL14. AFB1 exposure results in the reduction of m5C and m1A levels in both mRNA and tRNA, compromising RNA stability and translational accuracy [32]. Mechanistically, AFB1 binds to the catalytic site of methyltransferases, altering their conformation and reducing substrate binding affinity, which has been shown to lead to hepatic carcinogenesis in murine models [31].

#### 5-Methylcytosine (m5C) modification

A recent study has underscored the importance of m5C modification in RNA, especially in mRNAs [33]. This epigenetic modification affects RNA metabolism, stability, nuclear export, and translation [34]. The modification is essential in numerous physiological and pathological processes, including stress response, cancer, and embryogenesis [35]. m5C is subject to dynamic regulation by writers, erasers, and readers, and is present in several RNA types, including tRNAs, mRNAs, and rRNAs [34]. While TET enzymes (erasers) may help to demethylate, ALYREF and YBX1 proteins (readers) identify and control m5C-modified RNA function. DNMT2 and NSUN family enzymes (writers) catalyse m5C methylation [36]. Although less researched than m6A, m5C is gaining recognition as a significant epitranscriptomic marker with potential relevance for illness diagnosis, therapy, and monitoring Pesticides, industrial solvents, and endocrine disruptors like phthalates can dysregulate these enzymes, leading to aberrant m5C deposition [37]. Disruption of m5C impairs RNA export and translation and has been associated with immune suppression, metabolic dysregulation, and increased cancer risk [33]. For instance, phthalate-induced changes in m5C profiles in non-coding RNAs are linked to abnormal fetal development and adult-onset chronic diseases [38, 39]. Organophosphate pesticides such as chlorpyrifos and diazinon have been found to downregulate NSUN2, a key methyltransferase for m5C, especially in tRNAs and mRNAs [40]. This suppression leads to hypomethylation in detoxification-related genes, impairing translation fidelity and contributing to mitochondrial dysfunction, a central mechanism in neurodegenerative disorders. Organophosphates also induce A-to-I RNA editing errors through the inhibition of ADAR enzymes, which further destabilizes neuronal transcriptomes and synaptic plasticity [41]. Furthermore, the role of m5C in non-coding RNA regulation must be emphasized. Beyond mRNA, m5C is critical in small nuclear RNAs (snRNAs) and long non-coding RNAs (lncRNAs) [42]. Disruption of m5C in lncRNAs such as MALAT1 has been linked to altered chromatin structure and epigenetic regulation [43]. For example, phthalate exposure disrupts m5C in MALAT1 via downregulation of NSUN5, impairing its chromatin-binding function and thereby silencing tumor suppressor gene loci in urogenital tissues [44]. Moreover, m5C modifications in tRNAs affect codon–anticodon pairing, and their loss leads to ribosome stalling, increased frameshifting, and proteotoxic stress [44]. It is also essential to discuss the role of demethylases and erasers with specificity. For instance, ALKBH5 and FTO (Fat mass and obesity-associated protein) are the primary demethylases responsible for m6A removal [45]. Exposure to dioxins inhibits ALKBH5 activity through AhR (Aryl hydrocarbon receptor) pathway activation, leading to sustained m6A hypermethylation and aberrant gene expression profiles, particularly in immune cells. This contributes to T-cell dysfunction and heightened autoimmune risk [46].

#### Pseudouridine (Ψ) modification

Pseudouridine (Ψ) is a structural isomer of uridine that enhances RNA stability and ribosome function. Transcriptome-wide mapping approaches have identified Ψ sites in mRNAs and non-coding RNAs, broadening their recognised prevalence beyond tRNAs and rRNAs [47]. The Ψ alteration enhances RNA-RNA and RNA-protein interactions, hence affecting RNA stability and functionality [48]. It is associated with mRNA stability and translation efficacy, and Ψ-modified RNAs exhibit diminished innate immune responses, which is advantageous for mRNA vaccine development [48]. The pseudouridylation process, catalyzed by pseudouridine synthases (PUS), is not limited to structural RNAs like rRNAs and tRNAs but also extends to mRNAs, where it modulates splicing and translation. Tobacco-specific nitrosamines such as NNK (4-(methylnitrosamino)−1-(3-pyridyl)−1-butanone) reduce PUS1 expression and inhibit Ψ formation, destabilizing ribosomal RNAs and leading to inefficient translation [49, 50]. Experimental knockdown models confirm that this disruption contributes to defective ribosome biogenesis and proteostasis, key features in carcinogenesis [51]. Similarly, cadmium exposure significantly alters the activity of DKC1, a pseudouridine synthase that modifies both rRNA and snRNA. Inhibition of DKC1 compromises pre-rRNA processing and spliceosomal assembly, which is associated with ribosomopathies and myelodysplastic syndromes [52]. Thus, pseudouridine loss has cascading effects on both the transcriptome and proteome integrity [53]. Computational methods have been devised to effectively locate Ψ locations, augmenting experimental methodologies [54]. The identification of Ψ in mRNAs indicates a possible novel mechanism for proteome diversification and connects RNA pseudouridylation with cellular stress responses [47]. These findings underscore the increasing significance of Ψ modification in RNA biology and its prospective medicinal applications.

#### N1-Methyladenosine (m1A) modification

N1-Methyladenosine **(**m1A) is a common, reversible post-transcriptional alteration present in multiple RNA types, such as tRNA, rRNA, mRNA, and lncRNA [55]. (The alteration is dynamically controlled by “writers,” “erasers,” and “readers,” which are essential in RNA processing, structure, and function [56]. m1A has been demonstrated to influence gene expression regulation, cellular invasion, proliferation, and cell cycle regulation in cancer [56]. Research has concentrated on elucidating the role of m1A in cancer formation and progression, with prospective applications in diagnosis, therapy, and prognosis [56, 57]. Moreover, m1A levels in urine have been linked to illness development, indicating their potential as a biomarker for early detection and monitoring of numerous diseases, especially malignancies [58]. Exposure to bisphenol A (BPA) and other endocrine disruptors has been linked to altered m1A patterns, potentially affecting metabolic pathways [59]. Mechanistically, N1-methyladenosine (m1A) is regulated by TRMT6/TRMT61A complexes in mRNAs and by TRMT10C in mitochondrial tRNAs. Environmental endocrine disruptors such as Bisphenol A (BPA) directly impair TRMT6 expression, reducing m1A levels [58, 60]. This affects mitochondrial oxidative phosphorylation by disrupting mitochondrial tRNA folding and translation initiation [60]. This process results in ATP depletion and reactive oxygen species (ROS) accumulation, fueling inflammation and insulin resistance pivotal in the pathophysiology of obesity and type 2 diabetes. Moreover, m1A loss leads to reduced translation of anti-inflammatory mediators such as IL-10, which may contribute to the progression of chronic inflammatory diseases following environmental exposures [61]. Demethylases like ALKBH1 are also susceptible to toxicant inhibition, with exposure to mercury compounds shown to reduce their nuclear localization, leading to the buildup of aberrant m1A-modified RNAs [61].

#### 2′-O-Methylation (Nm) modification

Recent studies on 2’-O-methylation (Nm) in RNA underscore its ubiquity across diverse RNA types and its influence on physiological mechanisms. Writers such as fibrillarin catalyzes Nm modification. Nm changes impact RNA architecture, stability, and interactions, hence influencing translation, splicing, and the regulation of immunological responses [62, 63]. High-throughput detection techniques have progressed in NM research; nonetheless, hurdles persist in validating internal mRNA locations [62]. Nm has been associated with various diseases, including cancer, cardiovascular ailments, and neurological conditions [63, 64]. Research indicates that Nm can enhance the prevalence and duration of alternate RNA conformations, potentially modifying biological functions [65]. The therapeutic potential of Nm in RNA medicine is under investigation, with advancements in detection techniques like AI-based prediction and nanopore sequencing [64]. Viral infections and heavy metal exposure may disrupt Nm patterns, leading to aberrant immune signaling [66]. The 2′-O-methylation (Nm) process, primarily carried out by fibrillarin (FBL), is sensitive to heavy metals and air pollutants [63, 67]. Arsenic exposure, for example, reduces FBL expression, impairing Nm modification in rRNA and compromising translational fidelity. Nm loss has been associated with ribosome stalling at structured mRNA regions, triggering unfolded protein responses (UPRs) and subsequent apoptosis in lung epithelial cells [68]. Interestingly, oxidative stress generated by PM2.5 stimulates the nuclear translocation of small nucleolar RNAs (snoRNAs), which act as guides for Nm modifications. However, over-activation of this pathway can lead to aberrant Nm methylation in regions not normally modified, generating cryptic transcripts and oncogenic isoforms [69]. This suggests a dual-edged role of Nm under environmental stress, potentially contributing to adaptive but also pathological translational reprogramming.

Collectively, these disruptions show how environmental toxicants can reprogram the RNA modification machinery, compromising RNA function and contributing to disease susceptibility. These modifications are not passive targets but dynamic regulators of cellular responses to environmental cues. Their alteration precedes and predicts many of the downstream pathologies observed in environmental diseases. Understanding these interactions not only provides mechanistic insight into how toxins exert their effects but also opens up new possibilities for early detection of exposure, risk assessment, and targeted therapeutic interventions. As research advances, these epitranscriptomic marks may serve as sensitive biomarkers for environmental toxicity, offering a real-time window into how external insults translate into molecular dysfunction. Furthermore, the reversibility of many of these modifications offers therapeutic potential for modulating RNA responses and restoring cellular health in toxin-exposed individuals.

#### Emerging RNA modifications in environmental exposure

While our primary focus has been on five well-characterized RNA modifications, it is important to recognize that the RNA epitranscriptome is far more diverse. Several lesser-known modifications are increasingly gaining attention for their roles in cellular stress responses and disease progression. Among these, etheno (ε) derivatives such as 1,N6-ethenoadenosine (εA) and 3,N4-ethenocytidine (εC) are noteworthy [70]. These modifications are formed through interactions with reactive aldehydes generated during lipid peroxidation or through cytochrome P450-mediated metabolism of environmental toxins like vinyl chloride and acrolein [71]. Their formation has been associated with miscoding events that may contribute to genomic instability, cancer initiation, and neurodegeneration.

These etheno adducts are particularly relevant in the context of environmental exposure to pollutants found in cigarette smoke, industrial solvents, and combustion by products [72]. Their presence in RNA can disrupt normal translation and splicing processes, leading to altered gene expression patterns. Because they result from oxidative stress and persistent damage, εA and εC are increasingly being explored as biomarkers of toxic insult and early indicators of disease [72]. Including these modifications in environmental health research adds a valuable dimension to our understanding of how toxins affect RNA integrity beyond conventional epigenetic changes.

Another emerging class involves waiocine-type base analogues, which, although less well-studied, are hypothesized to arise under enzymatic stress conditions or toxic exposures [73]. Preliminary findings suggest they may be involved in adaptive cellular responses or pathological signaling in chronic exposure scenarios [73]. Advances in detection technologies such as mass spectrometry and nanopore sequencing are beginning to shed light on these rare modifications, opening new avenues for research. Expanding our view to include these non-canonical RNA modifications is essential for developing a more comprehensive understanding of RNA-mediated toxic responses and identifying novel biomarkers for environmental exposure and disease risk.

In addition to these rare modifications, some abundant RNA base modifications like methylguanosine (m7G) and 5-methylcytidine (m5C) have also been associated with pathological states [74]. These modifications occur naturally in transfer RNAs (tRNAs), ribosomal RNAs (rRNAs), and messenger RNAs (mRNAs), where they influence RNA stability, splicing, and translation. However, under abnormal physiological conditions or environmental stress, their levels and distribution may become dysregulated. For instance, elevated m5C levels have been linked to tumor progression and chemoresistance in various cancers, while aberrant m7G methylation patterns have been observed in neurological disorders and inflammatory diseases [75]. Their widespread presence and measurable alterations under stress make them promising epitranscriptomic markers of disease and exposure, further supporting the need to explore both rare and common RNA modifications in environmental health research.

### Techniques for detecting RNA modifications

The detection of RNA modifications is essential for understanding how environmental toxins alter RNA structure and function, ultimately leading to disease. With over 170 RNA modifications identified to date, researchers have developed a variety of biochemical, sequencing-based, and mass spectrometry methods to map, quantify, and analyze these modifications at both the transcriptome-wide and single-nucleotide levels [76, 77]. Each method has unique strengths and limitations and is often selected based on the specific RNA modification, desired resolution, and biological context.

#### Antibody-Based enrichment and sequencing (e.g., MeRIP-Seq, m6A-Seq)

This is one of the most commonly used techniques for detecting RNA modifications, particularly m6A [78, 79]. In this method, antibodies that specifically recognize modified bases (such as m6A) are used to immunoprecipitate the methylated RNAs, which are then subjected to high-throughput sequencing. This generates a transcriptome-wide map of RNA regions enriched in m6A [22, 80]. Techniques like MeRIP-Seq and m6A-Seq are widely applied in studies exploring environmental toxin exposure, as they allow for broad-scale identification of methylation patterns in response to stress [81]. However, one limitation is their resolution these methods typically pinpoint modified regions within ~ 100 nucleotides, but not exact sites. The technique is dependent on antibody specificity, which may affect sensitivity and reproducibility [81]. Nonetheless, due to its robustness and scalability, MeRIP-Seq has been extensively used to reveal changes in m6A distribution in diseases such as cancer and neurodegeneration caused by exposure to toxins like arsenic, PM2.5, or dioxins [82, 83]. The method is also compatible with both coding and non-coding RNA and can be used to assess dynamic changes over time. Its widespread use continues to support the discovery of toxin-responsive epitranscriptomic alterations and disease-linked m6A signatures.

#### Mass Spectrometry (LC-MS/MS, MALDI-TOF)

Liquid Chromatography–Tandem: Mass Spectrometry (LC-MS/MS) is a powerful tool for the detection and quantification of multiple RNA modifications at the nucleoside level, including m6A, m5C, Ψ, and 1-methyladenosine (m1A) [84]. It works by enzymatically digesting RNA into its constituent nucleosides, which are then separated based on their mass-to-charge ratio [84]. This allows for the precise identification and quantification of modifications, even at low abundance. LC-MS/MS is especially valuable in environmental toxicology because it provides absolute quantification and can reveal global shifts in RNA modification levels in response to toxin exposure [85, 86]. MALDI-TOF MS (Matrix-Assisted Laser Desorption/Ionization Time-of-Flight) is another mass spectrometry method offering high-throughput screening of RNA modifications [87]. These techniques are highly accurate and sensitive but do not provide sequence context; they cannot identify the exact nucleotide or transcript bearing the modification. Moreover, they require sophisticated equipment and trained personnel [88, 89]. Despite these challenges, MS-based tools are crucial for validating high-throughput sequencing results and detecting subtle changes in modification levels due to exposure to environmental pollutants like cadmium, bisphenol A, or lead [90]. The ability to quantify changes in RNA epigenetic marks makes MS indispensable in biomarker development and toxicological risk assessment.

#### Chemical conversion and sequencing (e.g., bisulfite sequencing, CMCT Mapping)

This class of detection methods relies on selective chemical treatment of RNA to induce specific changes in modified bases, which can then be detected through reverse transcription and sequencing [91]. For example, bisulfite sequencing is widely used to detect m5C, as bisulfite converts unmethylated cytosine to uracil while leaving m5C unchanged. This enables base-resolution mapping of m5C sites [92]. Similarly, CMCT (N-cyclohexyl-N’-(2-morpholinoethyl)carbodiimide) treatment is employed for the detection of pseudouridine (Ψ) [93]. It modifies Ψ residues, resulting in characteristic reverse transcription stops that can be read out by sequencing. These chemical-based techniques are useful for modification-specific studies and offer high positional accuracy. However, they can be labor-intensive, require careful optimization, and are not amenable to all modification types [94]. In addition, chemical treatments may introduce artifacts or yield incomplete reactions, potentially affecting reliability. These methods have been effectively applied in studies where exposure to pollutants such as aflatoxins, phthalates, or mercury disrupts methyltransferase activity, leading to hypomethylation or pseudouridine loss [94]. Because these modifications affect RNA stability and translation, accurate detection is essential for linking environmental exposures to downstream disease outcomes. Therefore, chemical conversion techniques remain a cornerstone in mechanistic epitranscriptomic research.

#### Direct RNA sequencing (Nanopore Technology)

Nanopore sequencing has emerged as a revolutionary tool in the field of epitranscriptomics by enabling direct RNA sequencing without the need for reverse transcription or amplification [95]. This technique works by passing native RNA molecules through a biological nanopore, where the electrical current changes based on the sequence and chemical modifications of the RNA. RNA modifications such as m6A, m5C, and Ψ alter the ionic current in distinct patterns, allowing them to be inferred computationally [96, 97]. The key advantage of nanopore sequencing lies in its ability to detect modifications in real time and at the single-molecule level. It can also sequence full-length RNA transcripts, preserving context and structural information. Despite its novelty, challenges include relatively lower base-calling accuracy compared to other sequencing platforms and the need for machine learning algorithms to improve modification detection [98]. This technology is increasingly applied in environmental epigenomics, where changes in RNA modifications can be detected in cells or tissues exposed to toxins like PM2.5, BPA, or heavy metals [98]. The portability, real-time capability, and single-molecule resolution make nanopore sequencing an invaluable asset in field-based environmental monitoring, as well as in clinical diagnostics [99].

#### CRISPR-Based epitranscriptomic biosensors

Emerging genome-editing technologies such as CRISPR-Cas13 systems are being re-engineered to detect and modify RNA modifications with unprecedented precision [100]. Specifically, dCas13 (deactivated Cas13) fused with either methyltransferases or demethylases enables targeted editing or detection of RNA epitranscriptomic marks like m6A and m5C [101]. These CRISPR-based biosensors can be designed to recognize specific RNA sequences and their modified forms, allowing real-time monitoring of environmental toxin-induced epitranscriptomic changes in living cells. One major advantage of this system is its programmable nature, which enables researchers to direct the system to virtually any transcript of interest. It also allows for dynamic tracking and manipulation of RNA modifications without altering the underlying genome [102, 103]. Applications of this technique include detection of environmental pollutants such as pesticides, heavy metals, and endocrine-disrupting chemicals that modulate methylation enzyme activity [102]. Although still in its early stages, CRISPR biosensor platforms show promise for use in point-of-care diagnostics and field-deployable biosurveillance tools. However, challenges remain, including potential off-target effects, delivery limitations, and the need for enhanced specificity [104]. Nonetheless, the adaptability of CRISPR-Cas systems offers a powerful approach for environmental toxicogenomics, mechanistic studies, and even the development of epitranscriptomic-based therapeutics.

#### Ribose-Seq and related techniques for 2′-O-Methylation (Nm)

Ribose-seq is a specialized chemical sequencing method designed to detect 2′-O-methylation (Nm), a key RNA modification that influences translation, splicing, and immunological responses [62]. This technique capitalizes on the resistance of Nm-modified nucleotides to alkaline hydrolysis or specific nucleases, enabling the mapping of Nm sites across the transcriptome [62, 105]. After hydrolysis, unmodified ribonucleotides are degraded, while Nm-modified residues remain intact and are sequenced to determine their exact positions. Ribose-seq, often used in combination with other tools like LC-MS/MS or nanopore sequencing, provides both positional and quantitative data [15]. Because Nm is often found in rRNAs, tRNAs, and snRNAs, its dysregulation by environmental toxins like arsenic, mercury, and PM2.5 has major implications for ribosome biogenesis, translational fidelity, and immune signaling. Importantly, under environmental stress, Nm modifications may become aberrantly distributed, leading to ribosome stalling, unfolded protein responses, and even oncogenic isoform formation. Ribose-seq and related assays such as Nm-Seq and RTL-P (Reverse Transcription at Low deoxy-ribonucleotide concentrations) are essential tools for understanding how such disruptions contribute to disease [106]. Despite technical challenges in validating internal Nm sites, these tools are advancing the field by uncovering nuanced RNA-level changes linked to environmental exposure.

#### Pseudo-Seq and Ψ-Seq for Pseudouridine mapping

Pseudouridine (Ψ) is the most abundant RNA isomerization modification and plays a pivotal role in RNA stability and function [107]. Techniques such as Pseudo-seq and Ψ-seq are developed to identify Ψ sites with high resolution across the transcriptome. These methods utilize chemical probes like CMCT (N-cyclohexyl-N’-(2-morpholinoethyl)carbodiimide) that selectively bind to Ψ residues [108]. Following treatment, the reverse transcription step results in characteristic termination or misincorporation events at Ψ sites, which are then detected through sequencing. These techniques are instrumental in identifying Ψ not only in rRNA and tRNA but also in mRNA and non-coding RNAs, revealing a broader regulatory landscape. Environmental toxins such as cadmium, NNK (a tobacco-specific nitrosamine), and heavy metals have been shown to suppress pseudouridine synthases (e.g., PUS1, DKC1), leading to global reductions in Ψ levels [109, 110]. These reductions impair ribosome function, spliceosome assembly, and protein synthesis, contributing to diseases such as cancer, neurodegeneration, and myelodysplastic syndromes. Pseudo-seq offers single-nucleotide precision and is particularly useful in evaluating toxin-specific transcriptomic disruption. Newer variations are incorporating high-throughput and machine learning approaches for more efficient data processing. As knowledge grows, Ψ mapping technologies will remain critical in evaluating how environmental exposures reshape the epitranscriptomic landscape.

### Functional roles of RNA modifications in cellular homeostasis

Recent studies underscore the essential function of RNA alterations in preserving cellular homeostasis and governing diverse biological activities. Post-transcriptional modifications, such as m6A, m5C, pseudouridine, and inosine, affect RNA stability, transport, and translational efficiency [111]. RNA alterations play a role in cell death, proliferation, senescence, differentiation, and metabolism [112]. They also assume context-dependent functions in stress responses, including oxidative stress, hypoxia, and DNA damage [112]. RNA changes are crucial for intricate physiological systems such as muscle and cardiac function, especially in mitochondrial activities [113]. Impairment of RNA-modifying enzymes can result in multiple diseases, such as cancer and neurological disorders [111, 112]. Comprehending the context-specific effects of these alterations may yield novel prospects for illness prevention and therapy by targeting these pathways [112].

## Environmental toxins as epitranscriptomic modulators

Environmental toxins are pervasive pollutants that interfere with cellular homeostasis through mechanisms such as oxidative stress, DNA damage, and metabolic disruption. Recent studies have revealed that these toxicants can also alter epitranscriptomic modifications, leading to dysregulated gene expression and disease development [4, 114]. Toxin-induced epitranscriptomic dysregulation can lead to abnormal gene expression, cell stress, apoptosis, and increased disease susceptibility, including cancer, metabolic diseases, and neurodegenerative disorders [7]. Table 1 is a summary of environmental toxins that alter RNA modifications, along with their underlying mechanisms, sources, and associated diseases.

**Table 1:** Key environmental toxins that alter RNA modifications, along with their underlying mechanisms, sources, and associated diseases

Toxin	RNA Modification Affected	Target RNA Species	Molecular Mechanism/Pathway	Source(s)	Associated Diseases	References
Arsenic	m6A	mRNA	Alters METTL3/METTL14 expression, dysregulates mRNA methylation, leading to abnormal gene expression	Contaminated water, pesticides, industrial exposure	Lung cancer, diabetes, cardiovascular diseases	[115, 116]
Cadmium	m5C in tRNA, RNA editing	tRNA, mRNA	Reduces NSUN2-mediated methylation, leading to translation errors and cellular stress	Tobacco smoke, contaminated food, industrial pollution	Kidney damage, osteoporosis, lung cancer	[117, 118]
Lead	m6A, pseudouridine (Ψ)	rRNA, ncRNA	Reduces m6A levels via FTO/ALKBH5 dysregulation; alters Ψ levels, impairing ribosome assembly	Lead paint, water pipes, batteries	Neurodevelopmental delay, hypertension	[28]
Mercury	RNA splicing defects via RBP disruption	pre-mRNA	Binds RNA-binding proteins (e.g., hnRNPs), causing alternative splicing errors	Fish, dental amalgam, industrial waste	Neurotoxicity, renal impairment	[119]
Polycyclic Aromatic Hydrocarbons (PAHs)	m6A, oxidative RNA lesions	mRNA	Induces ROS generation and METTL3 dysregulation, leading to altered methylation and transcript instability	Cigarette smoke, vehicle emissions, grilled meats	Lung cancer, CVD	[120]
Bisphenol A (BPA)	m6A, miRNA expression	mRNA, ncRNA	Suppresses METTL3 activity, downregulates m6A, impairs insulin signaling and β-cell function	Plastics, food can linings, receipts	Obesity, type 2 diabetes, reproductive issues	[121]
Dioxins	m6A, miRNA alterations	mRNA, miRNA	Activates AHR signaling, altering METTL14 expression and miRNA maturation	Waste incineration, contaminated food, herbicides	Immunotoxicity, cancers	[122]
Pesticides (e.g., organophosphates)	m6A, miRNA dysregulation	mRNA, miRNA	Alters FTO expression and miRNA targeting, contributing to mitochondrial dysfunction and oxidative stress	Agricultural chemicals, food residues	Parkinson’s disease, endocrine disruption	[123]
Per- and Polyfluoroalkyl Substances (PFAS)	m6A, miRNA expression	mRNA, miRNA	Dysregulates methyltransferases and demethylases, causing inflammation via IL-6/STAT3 pathway	Non-stick pans, firefighting foam, packaging	Liver damage, thyroid dysfunction, immune suppression	[124]
Airborne Particulate Matter (PM2.5, PM10)	m6A, RNA oxidation (8-oxoG)	mRNA	Increases ROS and affects METTL3/FTO, leading to pro-inflammatory gene activation	Vehicle exhaust, industrial smog	Asthma, COPD, cardiovascular diseases	[125, 126]
Mycotoxins (e.g., Aflatoxins)	m5C, m1A	tRNA, rRNA	Inhibits RNA methyltransferases (NSUN2, TRMT6), disrupting protein synthesis and detoxification	Moldy grains, nuts, dairy	Hepatocellular carcinoma, immune suppression	[127]
Phthalates	m5C in ncRNAs	lncRNA, miRNA	Alters NSUN2 and DNMT pathways, causing epigenetic changes during embryonic development	PVC, cosmetics, food containers	Reproductive toxicity, obesity	[128]
Polychlorinated Biphenyls (PCBs)	miRNA, m6A	miRNA, mRNA	Induces oxidative stress and alters miRNA expression affecting apoptosis and inflammation	Electrical equipment, industrial waste	Neurodegeneration, cancers	[129]
Asbestos	RNA oxidation, ncRNA dysregulation	lncRNA, miRNA	Generates ROS and modulates ncRNA expression involved in fibrogenesis	Building materials, insulation	Mesothelioma, lung fibrosis	[130]

### Mechanisms of action: how environmental toxins disrupt RNA modifications

Environmental toxins alter RNA modifications through the following key mechanisms:

i. Direct inhibition of RNA-modifying enzymes: Heavy metals such as arsenic and cadmium directly bind to methyltransferases (writers), reducing m6A and m5C methylation [131]. Dioxins interfere with demethylases (erasers), preventing RNA demethylation and leading to persistent transcriptomic changes [122]. Toxicants such as particulate matter, sodium arsenite, bisphenol A, and vinclozolin can drastically reduce m6A methylation levels in lung epithelial cells [11]. Exposure to high levels of PM2.5 has been linked to increased expression of RNA modification enzymes, specifically m6A writers, erasers, and readers [11]. Pesticides and endocrine disruptors alter the expression of RNA-modifying enzymes, leading to aberrant RNA methylation patterns [132]. Phthalates disrupt the cross-talk between RNA modifications and DNA methylation, leading to long-term epigenetic alterations [133].
ii. Induction of oxidative stress and RNA damage: Reactive oxygen species (ROS) generated by pollutants oxidize RNA bases, leading to modification errors and degradation [134]. Oxidative stress inhibits RNA-binding proteins (readers), disrupting translation and protein synthesis [135]. m6A, one of the most prevalent RNA modifications, has been demonstrated to be especially susceptible to environmental exposures, with m6A levels influencing disease development [136].

**Fig. 1:**
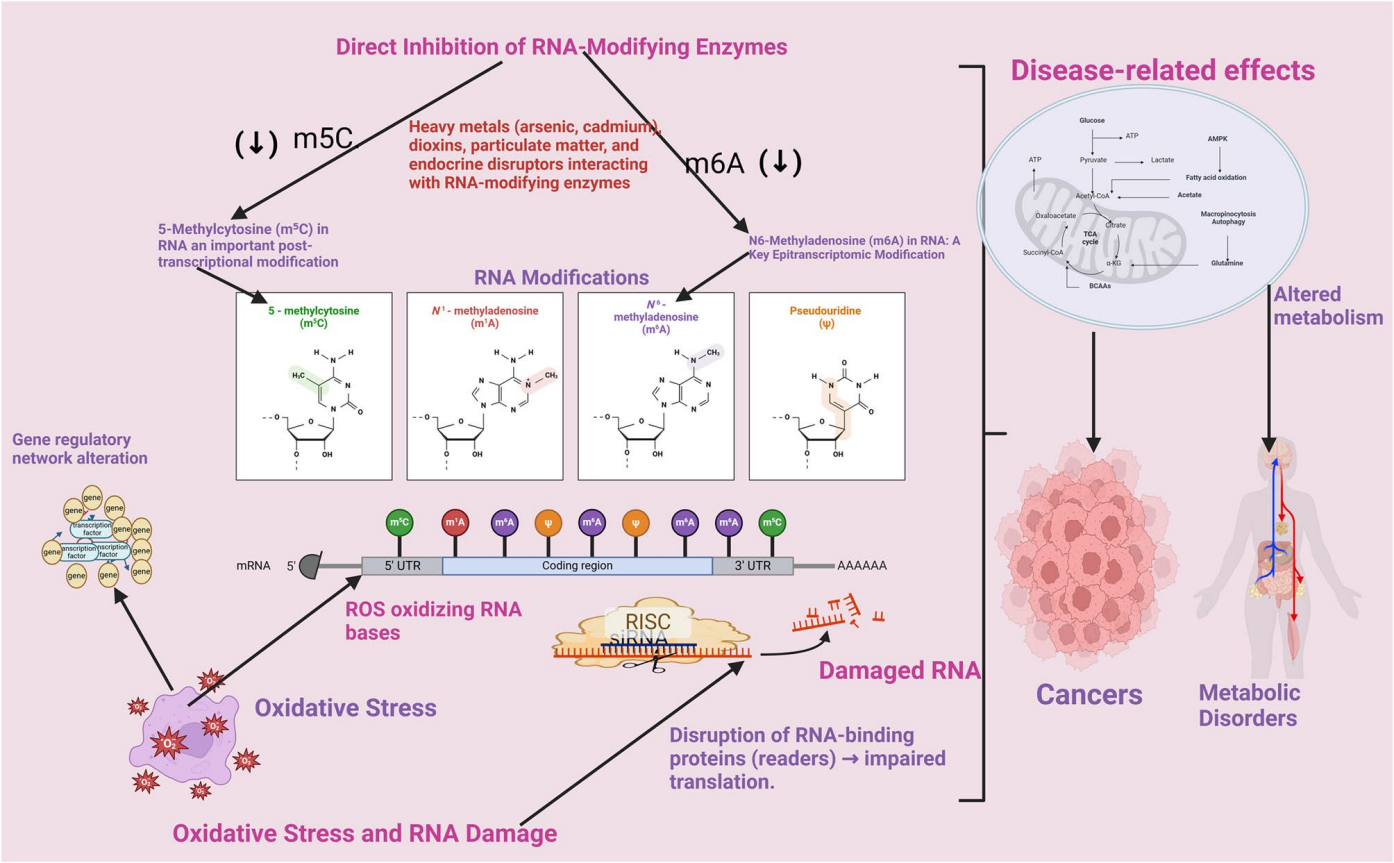
Environmental Toxins and RNA Modifications: Mechanisms of Alteration. (Created in https://BioRender.com)

Figure 1 illustrates the key molecular mechanisms through which environmental toxins disrupt RNA modifications. It highlights two primary pathways: (1) direct interference with RNA-modifying enzymes and (2) induction of oxidative stress that alters RNA integrity. Heavy metals such as arsenic and cadmium directly inhibit “writer” enzymes like METTL3 and NSUN2, impairing methylation processes (e.g., m6A, m5C). Simultaneously, air pollutants and pesticides trigger reactive oxygen species (ROS) generation, damaging RNA bases and destabilizing RNA-protein interactions. This dual disruption compromises RNA splicing, translation, and stability, leading to aberrant gene expression and disease development. The figure also maps the specific types of RNA modifications affected and the associated toxicants, providing a visual summary of how these pathways contribute to disease pathogenesis. By capturing these interactions, the diagram underscores the central theme of the review: RNA modifications act as molecular sensors and mediators of environmental stress with direct implications for human health.

## Epitranscriptomic biomarkers in environmental toxicology and disease diagnostics

With growing evidence that environmental toxins induce epitranscriptomic alterations, there is increasing interest in using RNA modifications as biomarkers for toxicant exposure, early disease detection, and prognosis. Traditional biomarkers, such as DNA mutations or protein markers, often detect disease at advanced stages. In contrast, RNA modifications are dynamic and reflect early cellular responses to toxicant exposure, making them promising indicators of environmental toxicity and disease susceptibility [137]. Environmental exposure can be measured through various biomarkers. Heavy metal exposure can lead to reduced m6A levels, increasing the risk of bladder cancer [138]. Lead toxicity disrupts m6A patterns in neural transcripts, linked to cognitive deficits in children [139]. Cadmium exposure induces m6A hypermethylation in lung epithelial cells, a potential marker for occupational lung disease [140]. Air pollution and pesticide exposure can also affect m5C and m1A levels, with reduced m5C levels linked to chronic exposure to PM2.5 and increased cardiovascular risk [141]. Pseudouridine (Ψ) dysregulation in tobacco smoke can impair protein synthesis and liver cancer risk [50]. Non-coding RNA modifications can predict metabolic disease, with tRNA-derived fragments with altered m5C modifications emerging as biomarkers for diabetes [142]. Recent studies underscore the promise of epitranscriptomic markers as diagnostic instruments. MicroRNAs (miRNAs) have emerged as promising biomarkers owing to their accessibility, specificity, and sensitivity [143]. RNA modifications, especially N6-methyladenine (m6A), are increasingly recognised for their regulatory functions in cellular pathways and disease mechanisms, indicating prospective applications in glioma diagnosis [144]. Adenosine-to-inosine (A-to-I) RNA editing patterns, governed by adenosine deaminase acting on RNAs (ADARs), are under investigation as diagnostic biomarkers for several disorders [145]. The domain of epitranscriptomics is progressing swiftly, presenting prospects for the identification of biomarkers and the development of therapeutic agents. Epitranscriptomic methodologies possess the capacity to revolutionise future healthcare frameworks, especially in illness diagnosis and prognosis [146]. As methodologies advance, epitranscriptomic markers are anticipated to be essential in personalised medicine and targeted therapeutics. Table 2 below summarises different RNA modifications that serve as biomarkers, their associated environmental toxins, detection methods, and disease implications.

**Table 2:** Epitranscriptomic biomarkers of environmental toxicants: detection methods, target rnas, and disease mechanisms

RNA Modification	Specific Environmental Chemicals	Target RNA Species	Detection Method	Downstream Molecular Pathways	Associated Disease(s)	References
m6A	Arsenic, Cadmium, Lead, Diesel Exhaust Particles	mRNA	LC-MS/MS, MeRIP-Seq, Nanopore sequencing	Dysregulation of METTL3/14; altered mRNA stability and translation efficiency	Cancer (lung, liver), neurodegeneration (AD), insulin resistance	[147]
m5C	Chlorpyrifos, Dioxins, Vinyl chloride	mRNA, tRNA, rRNA	Bisulfite sequencing, Nanopore sequencing, LC-MS/MS	Impaired NSUN2 activity; translation defects, stress response disruption	Immune dysfunction, cardiotoxicity, fatty liver disease	[148]
Ψ	PM2.5, Benzo[a]pyrene, Tobacco Smoke	rRNA, tRNA, snRNA	Pseudo-seq, Mass spectrometry	Disruption of PUS enzymes; decreased RNA stability and protein translation fidelity	Inflammatory diseases, lung cancer, COPD	[149]
N1-Methyladenosine (m1A)	BPA, Phthalates, PAHs	mRNA, tRNA	m1A-Seq, Mass spectrometry, HPLC	Methylation interference in tRNA decoding; metabolic pathway disruption	Obesity, reproductive disorders, endocrine dysfunction	[150]
Adenosine-to-Inosine RNA Editing (A-to-I Editing)	Lead acetate, Permethrin, PM2.5	mRNA, Alu repeats	RNA-Seq, ADAR assays, qPCR	Disrupted ADAR1/2 editing; neuroinflammation, altered neural gene expression	Autism spectrum disorders, epilepsy, MS	[151]
2′-O-Methylation (Nm)	Mercury, Arsenic, Trichloroethylene	rRNA, mRNA	LC-MS/MS, Ribose-seq, Mass spectrometry	Fibrillarin dysregulation; impaired ribosomal fidelity, immune gene silencing	Parkinson’s disease, lupus, asthma	[152]
N4-Acetylcytidine (ac4C)	Diesel soot, PM10, Vinyl acetate	mRNA, rRNA	LC-MS/MS, ac4C-Seq	NAT10 inhibition; altered mRNA stability, chromatin remodeling	Lung cancer, immune escape, leukemogenesis	[153]
2′-O-Methylguanosine (Gm)	PCBs, Mercury chloride, Aflatoxins	tRNA, rRNA	HPLC, LC-MS/MS	Altered TRMT112/FTSJ1 activity; stress response modulation	Autism, diabetes, immune suppression	[154]
tRNA-Derived Fragments (tRFs)	Arsenic, Glyphosate, DDT	tRNA	Small RNA-Seq, Northern blot	Induction of angiogenesis, inflammation via tRF-Gly-GCC, oxidative stress	Atherosclerosis, obesity, type 2 diabetes	[155]
N6,2′-O-Dimethyladenosine (m6Am)	Dioxins, Benzene, PM2.5	mRNA (5′ cap)	LC-MS/MS, MeRIP-Seq	FTO demethylase disruption; mRNA cap-dependent translation dysregulation	COPD, lung cancer, chronic bronchitis	[156]

### Advantages of epitranscriptomic biomarkers over traditional biomarkers

The following are key advantages of epitranscriptomic biomarkers over traditional methods:

#### Early detection

One of the most significant advantages of epitranscriptomic biomarkers is their capacity for early detection of disease or cellular dysfunction. RNA modifications, such as m6A, Ψ, and m5C, are dynamic and sensitive to environmental changes [53]. They respond rapidly to various stressors, including environmental toxins, oxidative stress, and chemical exposures, even before phenotypic or physiological symptoms manifest [53]. This sensitivity enables researchers and clinicians to identify molecular alterations at the earliest stages of disease progression, well before traditional biomarkers such as protein levels or histopathological changes can be observed.

Furthermore, epitranscriptomic changes often occur in a reversible and tightly regulated manner, allowing them to serve as real-time indicators of ongoing cellular processes [157]. Their presence can offer a window into disrupted gene expression patterns, aberrant cellular signaling pathways, and stress responses that precede irreversible damage or clinical symptoms. As a result, using RNA modifications as biomarkers has the potential to significantly improve diagnostic timelines, allowing for earlier therapeutic intervention and better patient outcomes. In contrast, conventional biomarkers frequently detect disease at later stages, when cellular damage may be more extensive and harder to reverse, limiting the effectiveness of treatment [158].

#### Non-invasive sampling

One of the key advantages of epitranscriptomic biomarkers is their compatibility with non-invasive sampling methods [159]. Unlike traditional tissue biopsies, which are often painful, risky, and require clinical settings, epitranscriptomic biomarkers such as chemically modified RNAs can be detected in easily accessible biofluids including blood, urine, and saliva [160]. This allows for routine and repeatable sampling, facilitating early diagnosis, disease monitoring, and treatment evaluation without causing discomfort to the patient [160]. For instance, extracellular vesicles and circulating RNAs in plasma or serum often carry distinct RNA modifications that reflect physiological or pathological conditions in real-time [161]. Moreover, these biofluids are relatively easy to obtain and store, making them ideal for longitudinal studies and large-scale screenings. The ability to track disease-associated RNA modification patterns through minimally invasive means represents a significant advancement in personalized medicine and public health, particularly for monitoring exposure to environmental toxins and early-stage disease processes [162].

High specificity and sensitivity

One of the most compelling advantages of epitranscriptomic biomarkers is their exceptional specificity and sensitivity in detecting environmental exposures. Certain RNA modifications such as m6A, m5C, and Ψ are selectively altered in response to specific environmental toxins like heavy metals, air pollutants, pesticides, and endocrine-disrupting chemicals [163]. These modifications act as unique molecular signatures, reflecting both the type and intensity of toxic exposure. Because these changes occur at the nucleotide level and often precede phenotypic alterations or disease onset, they offer a highly sensitive means of detecting early biological responses to toxic insults [164]. Additionally, the ability to link specific RNA modifications with particular chemicals or classes of toxins allows researchers and clinicians to trace the origin of exposure with great precision. This makes epitranscriptomic profiling a valuable tool not only for early detection but also for environmental risk assessment and personalized health monitoring [165].

#### Potential for real-time monitoring

One of the key advantages of epitranscriptomic biomarkers lies in the remarkable technological advancements in nanopore sequencing and mass spectrometry, which now enable real-time monitoring of RNA modification patterns [166]. Unlike traditional RNA sequencing methods that often overlook post-transcriptional modifications, nanopore sequencing can directly detect native RNA molecules without the need for reverse transcription or amplification [167]. This allows researchers to identify and quantify RNA modifications such as m6A, m5C, and Ψ in real time, across the entire transcriptome. Similarly, mass spectrometry-based approaches provide high-resolution, quantitative insights into the chemical structure of RNA modifications, offering precise identification and localization of modified nucleosides [168]. These methods have become increasingly sensitive and rapid, allowing dynamic assessment of how RNA modifications change in response to environmental stressors, toxins, or disease states. Together, these technologies empower scientists to capture transient and context-specific RNA modification events that are crucial for understanding regulatory mechanisms and disease pathogenesis. This real-time capability makes epitranscriptomic biomarkers highly promising for early diagnosis, monitoring of treatment efficacy, and personalized medicine [146].

### Epitranscriptomic regulation of DNA repair: implications for cancer progression and therapy resistance

Recent advances in epitranscriptomics have highlighted the functional significance of RNA modifications such as m6A, Ψ, and m5C in modulating DNA damage response (DDR) and repair pathways, thereby linking RNA metabolism to genomic stability [169]. These modifications serve not only as dynamic regulators of mRNA fate but also influence the recruitment and function of DNA repair proteins. For instance, m6A methylation is rapidly induced at sites of DNA double-strand breaks (DSBs), facilitating the recruitment of key repair factors like RAD51 and BRCA1, which are essential for homologous recombination (HR) [17]. This spatial and temporal regulation ensures efficient repair of damaged DNA and prevents accumulation of mutations, a critical safeguard against carcinogenesis. The enzymes responsible for adding (writers), removing (erasers), and recognizing (readers) these modifications such as METTL3, FTO, and YTHDC1 play critical roles in these processes, suggesting that epitranscriptomic regulators are integral to DDR networks [170].

In cancer, dysregulation of RNA modification machinery has emerged as a hallmark of tumor progression and therapy resistance [171]. Aberrant expression or mutations in m6A writers (e.g., METTL3/METTL14), erasers (e.g., ALKBH5/FTO), and readers (e.g., YTHDF1/2) disrupt the normal regulation of transcripts involved in cell cycle control, apoptosis, and DNA repair. For example, overexpression of METTL3 has been associated with enhanced DNA repair capacity in cancer cells, leading to resistance against genotoxic therapies such as chemotherapy and radiation [172]. Conversely, loss of m6A methylation can impair proper checkpoint activation and error-free DNA repair, fostering genomic instability. This dysregulation contributes to cancer initiation, progression, and poor clinical outcomes. Moreover, RNA modifications can fine-tune the expression of long non-coding RNAs (lncRNAs) and microRNAs that regulate DNA repair genes, amplifying their oncogenic potential [173].

The knowledge of the interface between RNA modifications and DNA repair provides a new avenue for therapeutic intervention in oncology. Targeting epitranscriptomic regulators may sensitize tumor cells to DNA-damaging agents or reverse resistance by impairing their adaptive repair mechanisms. For instance, inhibition of FTO or METTL3 has shown promise in preclinical models by disrupting DNA repair efficiency and promoting tumor cell death [174, 175]. Furthermore, profiling RNA modification patterns in tumors could serve as biomarkers for predicting treatment responses and stratifying patients for personalized therapy [176]. As the field progresses, integrating epitranscriptomics with genomic and proteomic data will be vital to unravel the complex crosstalk between RNA and DNA regulatory networks, ultimately offering new tools to combat cancer more effectively.

### Clinical and environmental applications of epitranscriptomic biomarker detection

Epitranscriptomic has emerged as a fundamental tool in biomarkers discovery in scientific discipline. The detection of these modifications, such as m6A, m5C, and Ψ, offers significant clinical and environmental applications. The availability of high-throughput sequencing technology together with mass spectrometry tools enables better identification of epitranscriptomic biomarkers which becomes fundamental to diagnostic tests and environmental detection systems [177]. Exploration of RNA modifications remains significant because it promises to create transformative medical achievements together with sustainable solutions.

#### Clinical applications

##### Cancer Diagnosis and Prognosis

The presence of abnormal RNA modifications leads to tumor formation and cancer cells advancing through different stages of disease. Altered m6A patterns in mRNA function as diagnostic and prognostic indicators in hepatocellular carcinoma and glioblastoma cancers [178].

##### Neurological Disorders

The modification patterns of RNA show disturbances in Alzheimer’s and Parkinson’s diseases which belong to the neurodegenerative disease category [179]. The detection of biomarkers in blood and cerebrospinal fluid through analysis may lead to the discovery of early symptoms for treatment purposes [180].

##### Infectious Disease Monitoring

The monitoring of infectious diseases includes epitranscriptomic modifications that occur between host and pathogen RNAs during SARS-CoV-2 and other viral infections. Research on particular RNA modifications serves to enhance antiviral strategy development and host response detection [181].

##### Personalized Medicine

RNA modification signatures can guide precision medicine by predicting patient responses to drugs, particularly in oncology and autoimmune diseases [182].

#### Environmental applications

Environmental Pollutants and Toxicology: Heavy metals, air pollutants, and endocrine disruptors exposure trigger epitranscriptomic changes in living organisms [183]. Monitoring these modifications in bioindicator species provides insights into environmental health. Microbial Community Analysis: Environmentally rich organisms with epitranscriptomic profiling of their microbiomes show adaptive trends that help scientists study both ecosystem modification research and pollution remediation efforts [184]. Agricultural and Food Safety: RNA modifications found in plants together with agricultural pathogens affect both crop resistance capabilities and pathogen resistance mechanisms [185]. Food quality assessment and health risks detection depends on detection techniques.

### Challenges and future perspectives of epitranscriptomic biomarker detection

The field of epitranscriptomics represents an emerging area of research that utilises RNA modifications to develop biomarkers used in disease detection including cancer, neurodegenerative and metabolic diseases. Multiple problems block the path to both detecting and implementing epitranscriptomic biomarkers within medical practice. Technological improvements along with improved analytical tools will help to achieve the complete potential of epitranscriptomics making it applicable for precision medicine and biomarker-based disease treatment.

#### Challenges

i. Lack of Standardized Protocols: The lack of uniform protocols for preparing samples along with identifying modifications while conducting analysis results in irreproducibility between studies [186]. Standardized protocols need to be established because they would enable result comparisons and biomarker validation. Clinical application requires standardization between sequencing and mass spectrometry approaches because of their current variability [187].
ii. Limited Availability of Robust Detection Tools: Robust detection tools available to study epitranscriptomics remain scarce due to their lack of precise resolution and mapping accuracy regarding modifications and their requirement for quantitative procedures [188].
iii. Interplay with RNA-Binding Proteins: RNA modifications create complex functional interpretations because they both affect and get affected by RNA-binding proteins (RBPs). The interpretation of these interactions needs improvements in multi-omics methodologies that continue to evolve [189].
iv. Clinical Translation and Validation: The clinical use of epitranscriptomic biomarkers remains limited because few such biomarkers have achieved successful validation in medical settings. The complexity involved in RNA modifications combined with diagnostic assay design difficulties and regulatory stockades obstructs clinical approval of these biomarkers [190]. The development of reference ranges for RNA modifications in health and disease requires additional cohort-based research.

#### Future perspectives

i. Advancements in Sequencing Technologies: Advance research on sequencing technology could lead to breakthroughs that would provide better resolution while detecting modifications instantly which can eads to precise biomarker recognition methods.
ii. Development of Targeted Detection Methods: The development of targeted detection methods including CRISPR-based and biosensor-based approaches that aim to deliver quick and cost-efficient specific detection of epitranscriptomic modifications.
iii. Integration with Artificial Intelligence and Machine Learning: The combination of artificial intelligence along with machine learning can enhance disease diagnostic accuracy by analyzing RNA modification patterns through large sequencing data collections.
iv. Multi-Omics Approaches: A complete biological understanding of RNA modifications at their biological significance can be achieved through the integration of epitranscriptomics with genomics together with transcriptomics, proteomics and metabolomics approaches.
v. Personalized Medicine Applications: Epitranscriptomic profiling could lead to personalized diagnostics and therapies, allowing tailored treatment strategies based on individual RNA modification patterns.

## Implications of epitranscriptomic alterations in disease pathogenesis

**Fig. 2:**
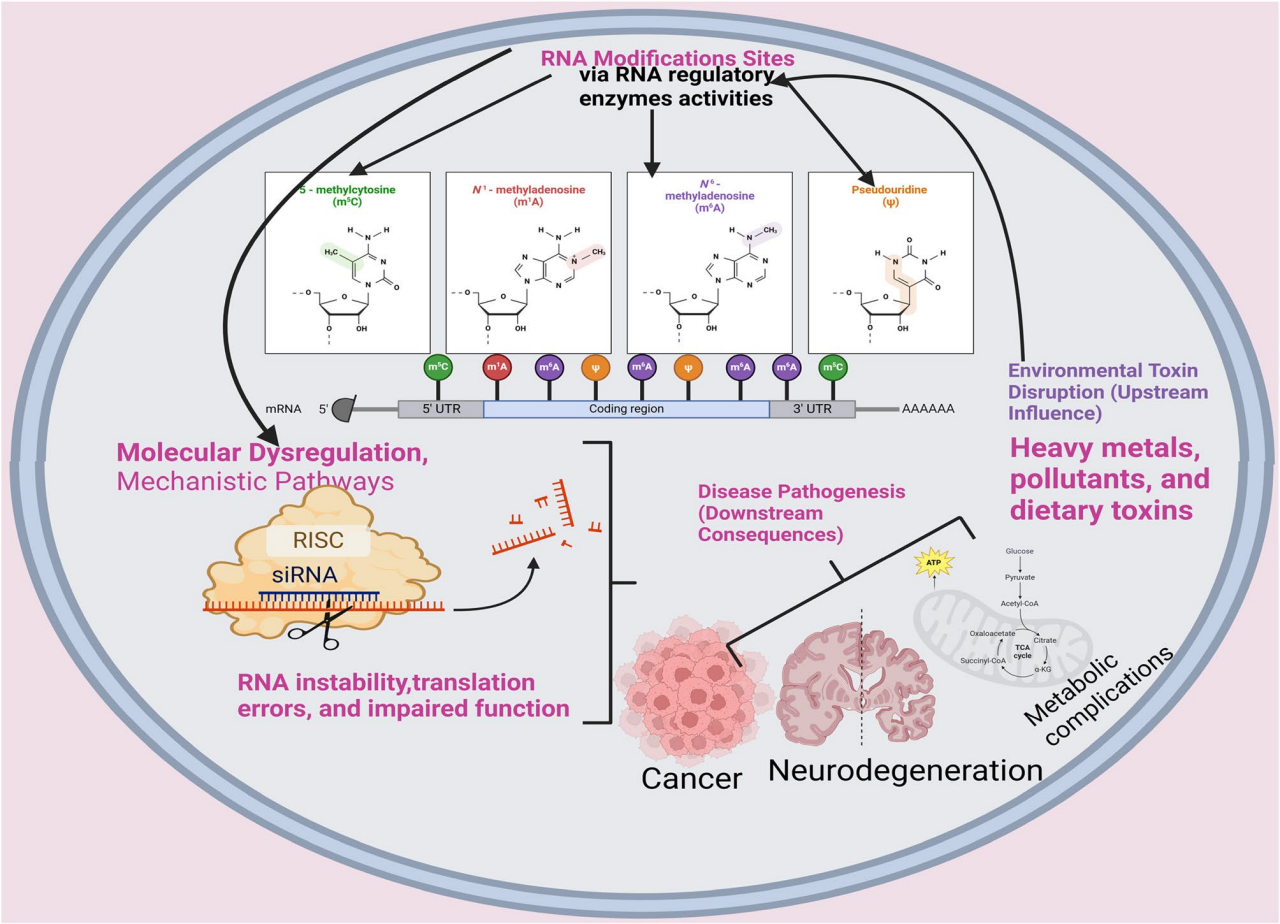
Implications of Epitranscriptomic Alterations in Disease Pathogenesis. (Created in https://BioRender.com)

Epitranscriptomic modifications play a fundamental role in maintaining cellular homeostasis by regulating RNA stability, translation, and function. Environmental toxins that disrupt these modifications contribute to molecular dysregulation, leading to various chronic diseases, including cancer, neurodegenerative disorders, metabolic syndromes, and cardiovascular diseases. Figure 2 below elucidates the pathogenic mechanisms linking toxin-induced RNA modifications to disease development. It presents an overview of how disrupted RNA modifications contribute to disease progression across multiple organ systems. It maps specific RNA marks (e.g., m6A, m5C, Ψ) to associated diseases such as cancer, neurodegenerative disorders, metabolic syndromes, cardiovascular conditions, and autoimmune diseases. For example, toxin-induced m6A dysregulation enhances oncogene translation in cancer, while altered pseudouridylation impairs protein synthesis in neurodegeneration. The figure also reflects how these epitranscriptomic changes affect critical cellular processes like inflammation, immune regulation, and mitochondrial function. Additionally, it highlights that many of these RNA alterations occur early in disease development, reinforcing their potential as predictive biomarkers. This visual summary supports the argument that environmental toxicants exert their pathogenic effects not just through genetic damage, but through dynamic and reversible changes in RNA chemistry. The figure integrates molecular disruption with systemic outcomes, emphasizing the clinical relevance of studying epitranscriptomic responses to environmental exposures.

### Cancer and tumor progression

RNA changes, especially m⁶A, are linked to many cancer-causing traits, such as tumour growth, invasion, stemness, and metabolic reprogramming [191]. These alterations impact many RNA species, including microRNAs, which are essential regulators of gene expression in cancer [192]. Epitranscriptomic alterations affect many phases of metastasis by modifying RNA structure and function; hence, they influence cellular processes essential for cancer dissemination [193]. The interaction between epigenetic and epitranscriptomic modifications influences all characteristics of human cancer, including dysregulations linked to advanced tumours [194]. Learning about these pathways could have big effects on preventing cancer, treating diseases that have spread to other parts of the body, and coming up with new ways to treat epitranscriptomic modifiers [191, 194]. Aberrant epitranscriptomic changes in response to environmental toxins help to initiate, spread, and resist therapy in cancer [195]. Arsenic exposure specifically downregulates METTL3, which reduces m6A methylation in tumor-suppressor RNAs, hence degrading them and fostering oncogenesis [196]. Similarly, cadmium-induced hypermethylation of m6A stabilizes carcinogenic mRNAs, promoting cell proliferation in lung and prostate tumors [197]. Dysregulated m6A-modified long non-coding RNAs (lncRNAs) increase chemoresistance in breast cancer and hepatocellular carcinoma [198]. Tobacco carcinogens also interfere with m5C modifications, which increases mRNA degradation in tumor-suppressor genes [53], while aflatoxin B1 exposure reduces translational fidelity, hence generating genomic instability and carcinogenesis [199].

### Neurodegenerative disorders

Heavy metal, pesticide, and air pollution neurotoxicants cause epitranscriptomic alterations that upset neural RNA homeostasis, hence aggravating neurodegenerative disorders including amyotrophic lateral sclerosis (ALS), Parkinson’s disease (PD), and Alzheimer’s disease (AD). RNA modifications such as m6A, m1A, m5C, pseudouridine, and A-to-I editing affect gene expression and cellular processes, playing a role in disease pathophysiology [17]. The epitranscriptome controls important parts of brain development and function. Neuropsychiatric, neurodevelopmental, and neurodegenerative diseases are linked to epitranscriptome dysregulation [200]. Epitranscriptomic modifications influence mRNA metabolism, splicing, export, localization, stability, and translation; hence, they affect essential physiological processes such as synaptic plasticity and neurogenesis [201]. The interaction of writers, readers, and erasers of RNA changes governs growth, health, and disease within the brain system [9]. Lead and mercury exposure reduces m6A changes in synaptic mRNAs, therefore compromising neural plasticity and memory capacity [202]. Pesticides reduce m1A methylation in mitochondrial tRNA, therefore causing poor ATP synthesis and neuronal death, a characteristic of Parkinson’s disease [203]. While pseudouridine loss in small nuclear RNAs (snRNAs) impairs RNA splicing in motor neurones, leading to ALS pathogenesis [204], cadmium toxicity changes m5C in tRNA, therefore decreasing protein synthesis and increasing the likelihood of β-amyloid and tau aggregation in Alzheimer’s disease [205]. Understanding these pathways could help researchers find new ways to treat neurodegenerative diseases. This shows how important it is to have advanced tools to track changes in RNA at both the genomic and single-molecule levels [17, 200].

### Metabolic disorders and diabetes

Environmental endocrine disruptors interfere with RNA modifications involved **in** insulin signaling, lipid metabolism, and energy homeostasis, leading to metabolic diseases. m⁶A alterations are recognised as essential in numerous disorders, including diabetes [206, 207]. Disruptions in the tRNA epitranscriptome, such as hypomodifications and fragmentation, have been linked to pancreatic β-cell dysfunction and diabetes [208]. The m6A and m6Am alterations, governed by writers, readers, and erasers, have been associated with diabetic tissues and may contribute to the pathogenesis of type 2 diabetes mellitus [207]. Mitochondrial RNA alterations have been associated with metabolic illnesses, indicating their role in mitochondrial-related diseases [209]. Bisphenol A and phthalates disrupt m6A levels in pancreatic β-cells, impairing insulin secretion and promoting type 2 diabetes [210]. Persistent organic pollutants induce m6A dysregulation in adipose tissue, altering lipid metabolism and contributing to obesity [211]. Again, heavy metals impair m1A-modified mitochondrial tRNAs, leading to defective oxidative phosphorylation and increased insulin resistance [212]. Pesticide exposure alters tRNA-derived fragments (tRFs), affecting glucose homeostasis and promoting metabolic syndrome [213]. These epitranscriptomic modifications provide prospective insights into the pathophysiology of diabetes and may facilitate the identification of innovative treatment targets and diagnostic instruments for metabolic illnesses [206, 209].

### Cardiovascular diseases

Epitranscriptomic alterations raise the risk of cardiovascular disorders by contributing to inflammation, atherosclerosis, and vascular dysfunction. RNA modifications, such as m6A, adenosine-to-inosine (A-to-I) editing, and m5C, are essential in governing RNA destiny and cellular functions [214, 215]. These alterations influence RNA decay, maturation, splicing, stability, and translational efficiency, presenting opportunities for innovative therapeutic strategies in cardiovascular diseases [216]. Epitranscriptomic modifications are especially significant in ischaemic cardiovascular disorders and atherosclerosis, as they affect critical processes such as cell proliferation [215]. RNA alterations exhibit a more responsive mechanism for environmental adaptation than genetic imprinting [216]. For instance, m6A hypermethylation in inflammatory mRNAs due to air pollution exposure (PM2.5) promotes endothelial cell dysfunction and atherosclerosis [217]. Likewise, cadmium disturbs m6A demethylation mediated by ALKBH5, which causes more vascular inflammation and hypertension [218]. M5C changes generated by heavy metals in non-coding RNAs promote macrophage foam cells and plaque formation [219]. Furthermore, m5C dysregulation in cardiomyocytes is associated to oxidative stress and myocardial infarction risk [220]. Given that cardiovascular disease continues to be a primary cause of death globally, investigating epitranscriptomic pathways presents valuable opportunities for enhancing early detection and formulating tailored therapeutics [215, 221].

### Autoimmune and inflammatory diseases

With environmental pollutants causing chronic inflammation and autoimmune, epitranscriptomic dysregulation is crucial in immune response modulation. m6A alteration has become a vital regulator of immune cell functionality and the progression of autoimmune diseases [222]. Epigenetic modifications, such as DNA methylation, histone alterations, and non-coding RNA expression, play a role in the pathogenesis of autoimmune and inflammatory illnesses throughout the inflammatory spectrum [223]. These alterations are especially significant in T cell development and malfunction in numerous disorders, including type 1 diabetes, rheumatoid arthritis, and systemic lupus erythematosus [224]. Dioxins and polychlorinated biphenyls change m6A-modified cytokine mRNAs, hence activating inflammatory pathways in lupus and rheumatoid arthritis [225]. Arsenic exposure alters tRNA methylation, therefore compromising T-cell function and raising autoimmune disease vulnerability [226]. Pesticides modify gut microbiota composition and cause gut inflammation in Crohn’s disease and ulcerative colitis, therefore affecting m5C-modified microbial RNA [227]. Transcriptomics has been essential in uncovering pathogenic signatures, novel therapeutic targets, and diagnostic and prognostic indicators in autoimmune and autoinflammatory disorders. This methodology has facilitated the identification of illness signatures mediated by IFN-, IL-1-, and IL-17, and is progressively employed for patient stratification and personalised treatment strategies [228].

### Transgenerational epitranscriptomic inheritance

According to Nilsson et al. [229], toxin-induced RNA changes can be passed on through generations, therefore affecting disease susceptibility. Environmental variables, senescence, nutrition, and exposure to toxicants can provoke epigenetic modifications in the germline, potentially resulting in heritable changes [230, 231]. These alterations encompass DNA methylation, histone changes, and non-coding RNAs, which can withstand genome-wide reprogramming events [230]. Transgenerational genetic effects have been documented, wherein genetic factors in one generation influence phenotypes in later generations without the direct transmission of the genetic variant [232, 233]. For instance, endocrine disruptors (e.g., BPA) alter sperm m6A/m5C signatures, thereby raising the metabolic disease risk in children [234]. Heritable tRNA and small RNA changes induced by heavy metals cause neurodevelopmental and immunological disorders across generations [235]. These findings complicate the identification of disease-related genes and may signify an adaptive strategy for conveying beneficial gene expression patterns between generations [233]. Understanding these mechanisms is essential for evaluating illness risk and investigating evolutionary consequences [230, 231].

## Therapeutic strategies targeting epitranscriptomic dysregulation

**Fig. 3:**
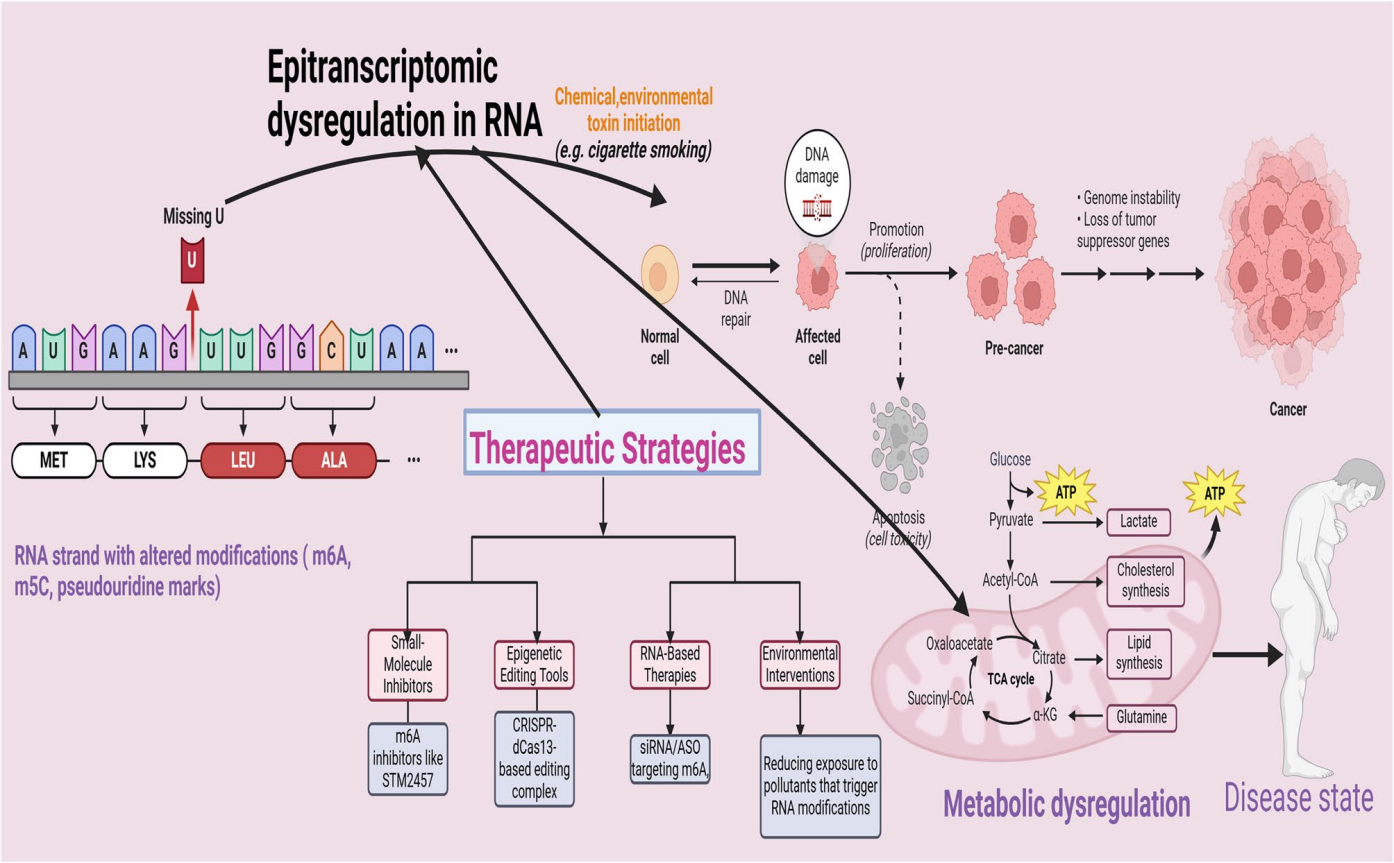
Therapeutic Strategies Targeting Epitranscriptomic Dysregulation (Created in https://BioRender.com)

The development of targeted therapies is urgently required because of the increasing evidence connecting environmental pollutants to epitranscriptomic alterations and disease emergence and progression. Approaches aimed at modulating RNA modifications include small-molecule inhibitors, epigenetic editing tools, RNA-based therapies, and environmental interventions **(**Fig. 3**).** Figure 3 illustrates emerging therapeutic approaches designed to counteract toxin-induced disruptions in RNA modifications. It categorizes strategies into three major domains: (1) small-molecule inhibitors that modulate the activity of RNA-modifying enzymes such as METTL3 or FTO; (2) CRISPR-based epitranscriptomic editing tools that allow precise correction of abnormal RNA marks; and (3) RNA-based therapeutics like modified oligonucleotides and RNA vaccines that leverage or mimic stable RNA modifications for therapeutic benefit. The figure also includes pathways illustrating how these interventions aim to restore RNA stability, normalize gene expression, and reduce toxin-induced cellular stress. By visualizing the link between molecular targets and treatment strategies, the figure emphasizes the translational potential of epitranscriptomic research. It reinforces the idea that RNA modifications are not merely passive markers of disease but actionable therapeutic targets. This aligns with the manuscript’s broader theme of integrating environmental toxicology with RNA biology to advance precision medicine and personalized intervention strategies.

### Small-Molecule modulators of RNA modifications

Small-molecule inhibitors and activators targeting RNA-modifying enzymes have shown promise in correcting aberrant RNA methylation patterns. Recent studies underscore the promise of targeting RNA alterations, specifically m6A, as a therapeutic approach in cancer treatment. Dysregulation of epitranscriptomic machinery proteins (EMPs) and modified RNA patterns have been associated with numerous illnesses, including cancer [236]. Small molecule inhibitors aimed at m6A regulatory proteins, including writers, erasers, and readers, are under development as prospective anti-cancer medicines [237]. Many tumours have been connected to too much METTL3 expression [238]. By lowering aberrant m6A methylation in carcinogenic transcripts, small-molecule METTL3 inhibitors including STM2457 have exhibited anti-leukemic benefits [239]. When active, m6A demethylases ALKBH5 and FTO restore RNA equilibrium in metabolic disorders including neurodegenerative ones [240]. Investigating FTO-targeting molecules for treatment of type 2 diabetes and obesity is in progress [241]. Other therapeutic targets include m5C and pseudouridine modulators. For instance, NSUN2 is a m5C methyltransferase whose malfunction is associated with neurological and cardiovascular diseases [242]. Small-molecule activators of NSUN2 could restore neuronal activity and guard against cognitive loss brought on by toxins [243]. Synthetic pseudouridine modifications improve RNA stability and thus utilized in mRNA medications including COVID-19 vaccines. One could use this strategy to offset RNA destabilization caused by toxins. Preclinical evidence substantiates the therapeutic promise of targeting these proteins, with initial human trials presently evaluating small molecule inhibition of the METTL3/METTL14 methyltransferase complex [244]. The domain of epitranscriptomics has progressed swiftly, propelled by enhanced methodologies for the characterization and quantification of RNA alterations [245]. Nonetheless, obstacles persist in the creation of efficient small molecule modulators and in converting these discoveries into practical advantages for cancer patients.

### CRISPR-Based epitranscriptomic editing

CRISPR-Cas technology has been adapted to directly edit RNA modifications, offering a precise and reversible approach to restore toxin-induced RNA dysregulation. CRISPR-Cas13 systems have been engineered for RNA editing, specifically targeting m6A alterations [246]. These instruments provide programmable RNA methylation and possess prospective applications in addressing disorders associated with epitranscriptomic modifications [236, 246]. Adenosine-to-inosine (A-to-I) RNA editing facilitated by adenosine deaminase acting on RNA (ADAR) proteins has demonstrated potential in rectifying disease-associated G-to-A point mutations [247]. The transient effects of RNA editing, in contrast to genome editing, provide it a compelling therapeutic strategy [246]. CRISPR-dCas13 combined with methyltransferases can add or remove m6A/m5C changes at specific sites, making them a possible treatment for cancer and neurological diseases [53, 248]. Systems of RNA-guided pseudouridine modification could help restore RNA stability in disorders related to environmental contaminants [249]. The fusion proteins of dCas9 with RNA methylation enzymes can precisely change the m6A and m5C marks, making them useful for targeted treatment of metabolic and cardiovascular diseases. The improvements in epitranscriptomic editing tools present novel opportunities for exploring the roles of RNA changes in stem cell differentiation, brain development, and numerous disease disorders [246, 250].

### RNA-Based therapeutics

RNA modifications and regulatory genes have surfaced as prospective therapeutic targets, with more than 170 types of alterations recognised in diverse RNAs [251]. Methods to regulate epitranscriptomic machinery proteins (EMPs) encompass small-molecule inhibitors, proteolysis-targeting chimaeras, and instruments for site-specific alteration of RNA [236]. Antisense strategies, including RNase H-dependent degradation, splicing correction, and siRNA interference, have demonstrated efficacy in the treatment of genetic diseases [252]. Innovative methods like as ADAR-mediated RNA editing, targeted pseudouridylation, and 2’-O-methylation provide alternatives to CRISPR-based technologies for rectifying genetic mutations [252]. RNA-based therapeutics are expected to broaden the spectrum of druggable targets beyond conventional small molecules and biologics [253]. Promising treatments against toxin-induced RNA alterations and related clinical effects arise from advancements in RNA-based medicines. For example, using pseudouridine in modified mRNA treatments enhances RNA stability and translation, thus offering possible treatments for metabolic disorders associated to environmental contaminants as well as neurological diseases [254]. mRNA-based treatments are being developed targeted at epitranscriptomic enzymes aiming to restore normal RNA activity in metabolic diseases and cancer [4]. More so, antisense oligonucleotides (ASOs) aiming at aberrantly altered mRNAs can break down harmful transcripts in neurological disorders and cancer [255]. Targeting RNA-modifying enzymes using siRNAs could help to control RNA methylation patterns and reverse epitranscriptomic changes caused by toxins [256]. Nonetheless, obstacles persist in the effective development and implementation of these medicines.

### Environmental and lifestyle interventions

Reducing exposure to environmental toxins while promoting nutritional and lifestyle interventions can help mitigate epitranscriptomic dysregulation. Environmental exposures, including benzo[a]pyrene, bisphenol A, and metals, can influence RNA alterations and the expression of their “writers,” “erasers,” and “readers” [257]. Epigenetic mechanisms, such as DNA methylation and histone changes, act as a conduit between the genome and environmental factors, significantly contributing to cancer formation and progression [258]. For heavy metals like mercury, cadmium, and lead, chelation therapy lessens their effect on RNA alterations. Green tea, curcumin, and resveratrol’s dietary antioxidants—polyphenols and flavonoids—counterbalance toxin-induced epitranscriptomic alterations [259, 260]. Methyl donors like folate and vitamin B_12_ help RNA methylation pathways, therefore lowering the effect of environmental pollutants [261]. Fish oil’s omega-3 fatty acids control inflammation and RNA integrity, therefore shielding against diseases brought on by toxins [262]. Lifestyle factors, nutrition, and psychological effects might impact epigenetic modifications, hence contributing to cancer and other disorders [263]. Natural epigenetic modifiers exhibit promise for cancer chemoprevention, connecting public health, environmental factors, and lifestyle choices [263]. These findings highlight the significance of environmental and lifestyle modifications in mitigating epitranscriptomic dysregulation and their potential as therapeutic approaches.

## Emerging technologies for studying epitranscriptomic alterations

With the advent of creative technologies allowing the detection, measurement, and manipulation of RNA modifications, the research of epitranscriptomic modifications in RNA is fast advancing. These emerging technologies are pivotal in understanding how environmental contaminants cause RNA changes and how these changes support disease pathogenesis. These technologies will surely result in the creation of novel therapeutic approaches meant to restore RNA homeostasis and reduce the effects of environmental toxin exposure as they continue to develop. Below are some tools and techniques designed to investigate epitranscriptomic modifications at the molecular level.

### High-Throughput sequencing technologies

Next-generation sequencing (NGS) has transformed the capacity to investigate RNA modifications at an unparalleled extent and enable the measurement of RNA alterations across the entire transcriptome.

i. m6A-Seq and MeRIP-Seq: m6A-seq method involves immunoprecipitation of m6A-modified RNA followed by high-throughput sequencing, providing a comprehensive view of m6A modification across the entire transcriptome [264]. Methylated RNA immunoprecipitation sequencing (MeRIP-seq) enables the detection of m6A methylation in RNA and has become an essential tool in understanding the regulation of gene expression in response to environmental toxins [200]. Both methods let scientists spot both global and specific patterns of RNA methylation, therefore offering understanding of how environmental stresses affect RNA structure and activity.
ii. Pseudo-Seq and m5C-Seq: Pseudo-Seq technique allows RNA to have pseudouridine alterations identified and quantified. Pseudo-seq offers important new perspectives on pseudouridine modification as a means of shielding RNA from breakdown because environmental contaminants might affect RNA stability and translation [265]. The m5C-Seq method helps to map m5C changes on RNA, therefore enabling an awareness of how methylcytosine alterations affect gene expression in response to environmental stresses [35]. These technologies taken together greatly help us to better grasp the several functions of RNA alterations in disease.

### CRISPR-Based epitranscriptomic editing

CRISPR-based tools have been adapted to target and edit RNA modifications, providing powerful methods for studying their functional roles in health and disease.

i. *CRISPR-associated protein 13* ***(***dCas13)-Based RNA Modifications: The dCas13 system allows for the precise and reversible modulation of RNA modifications in vivo. Target RNAs can have particular modifications, including m6A or m5C added or removed by combining dCas13 with RNA-modifying enzymes. This approach helps to functionally analyse the function of particular RNA alterations in the control of gene expression and disease progression [266, 267]. Direct evaluation of how toxins affect the alteration landscape of RNAs and how this affects cellular function can be accomplished with the dCas13 system.
ii. Epitranscriptomic Editing with CRISPR-Cas13f: Recent advances have led to the development of CRISPR-Cas13f, a smaller and more efficient version of Cas13, offering better delivery and greater precision [268]. Researchers are exploring CRISPR-Cas13f for epitranscriptomic editing with possible uses in targeted RNA modification treatments for diseases induced by environmental toxins [269].

### Mass Spectrometry-Based detection of RNA modifications

Emerging as a very sensitive technique for RNA modifications, mass spectrometry (MS) enables quantification and identification of specific changes with an uncommon accuracy.

i. Liquid chromatography-tandem mass spectrometry (LC-MS/MS) is principally useful for the identification of low-abundance RNA modifications including m5C, m6A, and pseudouridine [270]. Through this method, scientists may identify changes in particular areas of RNA, therefore offering a very thorough picture of how toxins affect RNA structure. High-resolution mass spectrometry also detects several changes in a single RNA molecule, providing hitherto unparalleled knowledge of the crosstalk between several RNA modifications and their possible synergy in control of gene expression in response to environmental contaminants [271].
ii. Another effective instrument for profiling RNA changes in complicated samples is matrix-assisted laser desorption/ionization-time of flight mass spectrometry (MALDI-TOF MS). This method provides high-throughput and effective detection of m6A, m5C, and other modifications, therefore allowing researchers to investigate environmental toxic effects on RNA modification [272].

### Single-Cell epitranscriptomics

The study of RNA modifications in individual cells is essential for understanding how environmental toxins affect cellular heterogeneity and disease progression. By means of epitranscriptomic profiling and single-cell RNA sequencing (scRNA-seq), RNA alterations in single cells can be analysed, therefore offering a comprehensive picture of cell-to-cell variability in response to environmental challenges [273]. By tracking variations in RNA modification over time, this approach offers insights on how individual cells cope with damage caused by toxins and how this affects the course of disease. The integration of scRNA-seq with epitranscriptomic methods is pivotal for the identification of particular cellular populations more sensitive to environmental toxins and possible development of new diagnostic biomarkers for disorders connected to toxic exposures [274].

### Nanotechnology for RNA modification detection

Nanotechnology is increasingly being used to improve sensitivity and specificity in the detection of RNA modifications, allowing for high-throughput analysis and real-time monitoring. RNA-based biosensors are developed to identify particular RNA alterations in real time. These biosensors sense changes in RNA modification patterns by means of nanomaterials like gold nanoparticles and graphene oxide [275]. They can be used to follow these changes across illness development and track how environmental contaminants impact RNA modifications in real time. Furthermore, very promising are nanoparticles in delivering tiny compounds or RNA-modifying enzymes to particular cells or tissues [276]. These developments might offer new strategies for cellular targeting and correction of toxin-induced RNA changes.

## Challenges and research directions

Although treatment approaches targeting at epitranscriptomic dysregulation show promise, several challenges still exist.

i. ***Delivery challenges***: An effective, tissue-specific delivery of RNA-based medicines is still a big challenge. Future studies should focus on creating safer and more efficient RNA-targeting treatments, improving delivery systems, and investigating new dietary and environmental strategies to reduce epitranscriptomic dysregulation brought on by toxins.
ii. ***Off-target effects***: Epitranscriptomic editing based on CRISPR could bring unplanned changes [277
iii. ***Long-term safety***: Entering the field of RNA editing begs issues of the long-term safety of changing RNA methylation patterns. Changing RNA methylation patterns has long-term effects that demand more research.
iv. ***Limited Standardized Protocols***: The dearth of thorough, uniform methods for identifying and quantifying RNA changes in clinical samples is a key issue. Developing more sensitive and high-throughput epitranscriptomic profiling technologies will be essential for large-scale studies to validate potential biomarkers and therapeutic targets.
v. ***Interindividual variability in response to environmental toxins***: Genetic and epigenetic elements could affect how each person reacts to hazardous exposures and how their RNA modifications are modified [278

## Conclusion

Epitranscriptomic modifications represent a fundamental regulating layer in RNA biology, influencing gene expression, cellular homeostasis, and disease progression. This study highlights the growing evidence that environmental toxins, including heavy metals, air pollutants, and endocrine disruptors, can cause notable changes in RNA modifications, leading to dysregulated cellular functions and raising disease vulnerability. Understanding toxin-induced epitranscriptomic alterations is essential since disruptions in important RNA modifications including m6A, m5C, and pseudouridine have been linked to cancer, neurological diseases, metabolic syndromes, and immunological dysfunction. Precision mapping and modulation of RNA changes made possible by recent advances in mass spectrometry, high-throughput sequencing, and CRISpen-based RNA editing will provide new paths for biomarker development and focused treatments. While small-molecule inhibitors and RNA-based therapies are possible approaches to minimise toxin-induced RNA dysregulation, the development of epitranscriptomic biomarkers has enormous promise for early illness detection and environmental toxicity assessment. Before clinical translation, nevertheless, issues including standardising detection techniques, off-target effects in RNA editing, and individual variability in toxin reactions have to be resolved. Integrating multi-omics techniques, enhancing single-cell epitranscriptomics, and creating individualised therapeutic strategies to counteract the negative consequences of environmental toxins should be the main priorities of further studies. Deepening our knowledge of epitranscriptomic control in environmental toxicology may open the path for creative diagnostic tools and treatment approaches protecting human health against toxin-induced disorders.

## Data Availability

No datasets were generated or analysed during the current study.
